# Supplementary material for: Discovery of Novel Boron-Containing N-Substituted Oseltamivir Derivatives as Anti-Influenza A Virus Agents for Overcoming N1-H274Y Oseltamivir-Resistant
Source: Molecules. 2022 Sep 29;27(19):6426. doi: 10.3390/molecules27196426 (PMC9571049; doi:10.3390/molecules27196426)

# Supporting information

# Design, Synthesis and Biological Evaluation of Novel Boron-Containing N-Substituted Oseltamivir Derivatives as Anti-influenza A Virus Agents for Overcoming Oseltamivir-Resistant N1-H274Y Variant

Ruifang Jia[a], Jian Zhang[b], Chiara Bertagnin[c], Anna Bonomini[c], Laura Guizzo[c], Jiwei Zhang[a], Zhen Gao[a], Zhuo Li[a], Chuanfeng Liu[a], Han Ju[a], Xiuli Ma[d], Arianna Loregian[c], Bing Huang[d,*], Peng Zhan[a,e*], Xinyong Liu[a,e*]

[a] *Department of Medicinal Chemistry, Key Laboratory of Chemical Biology (Ministry of Education), School of Pharmaceutical Sciences, Cheeloo College of Medicine, Shandong University, 44 West Culture Road, 250012, Jinan, Shandong, P.R. China*

[b] *Institute of Medical Sciences, The Second Hospital, Cheeloo College of Medicine, Shandong University, 250033*

[c] *Department of Molecular Medicine, University of Padova, via Gabelli 63, 35121 Padova, Italy*

[d] *Institute of Poultry Science, Shandong Academy of Agricultural Sciences, 1 Jiaoxiao Road, Jinan, Shandong, 250023, P.R. China*

[e] *China-Belgium Collaborative Research Center for Innovative Antiviral Drugs of Shandong Province, 44 West Culture Road, 250012 Jinan, Shandong, PR China.*

[*] *E-mails: hbind@163.com (Huang B.); zhanpeng1982@sdu.edu.cn (Zhan P.); xinyongl@sdu.edu.cn (Liu X.Y.)*

# The $^{1}$H NMR and $^{13}$C NMR spectra of compound 1c

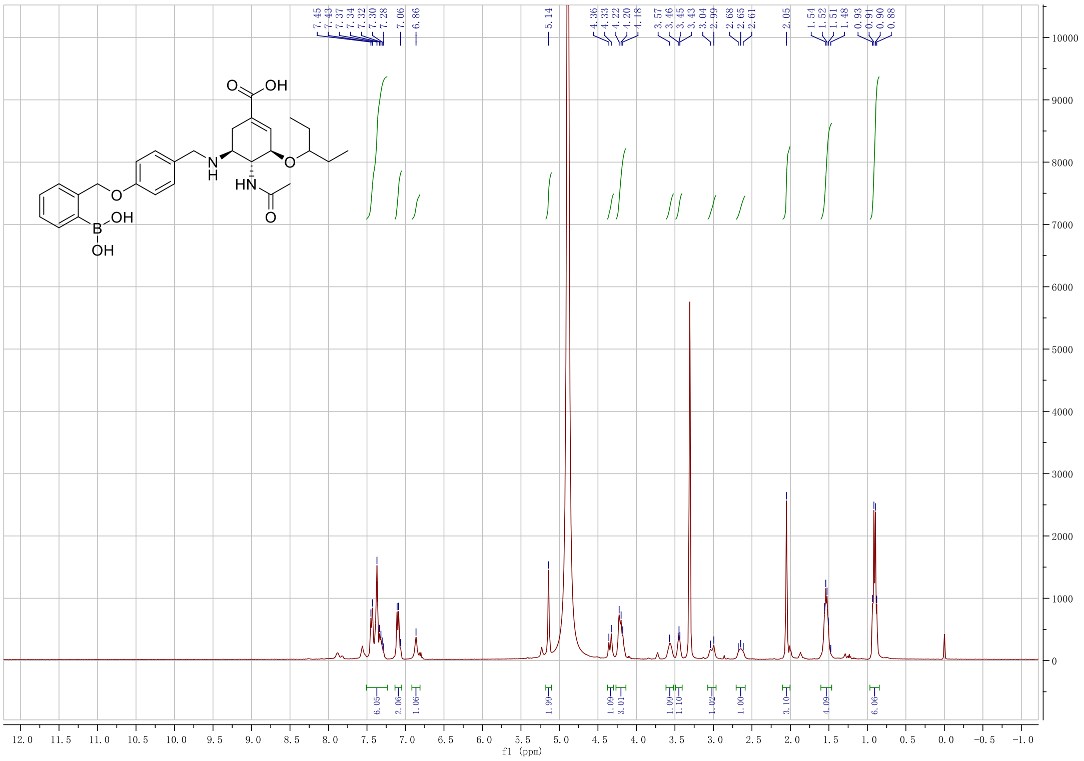

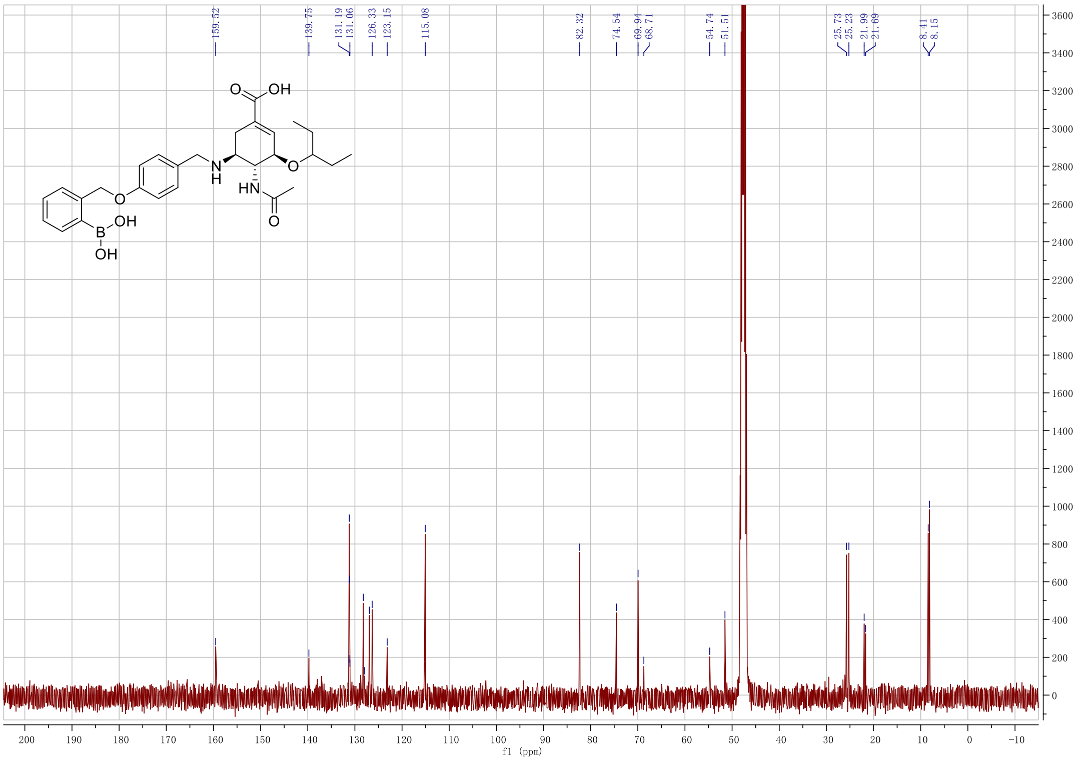

# The $^{1}$H NMR and $^{13}$C NMR spectra of compound 2c

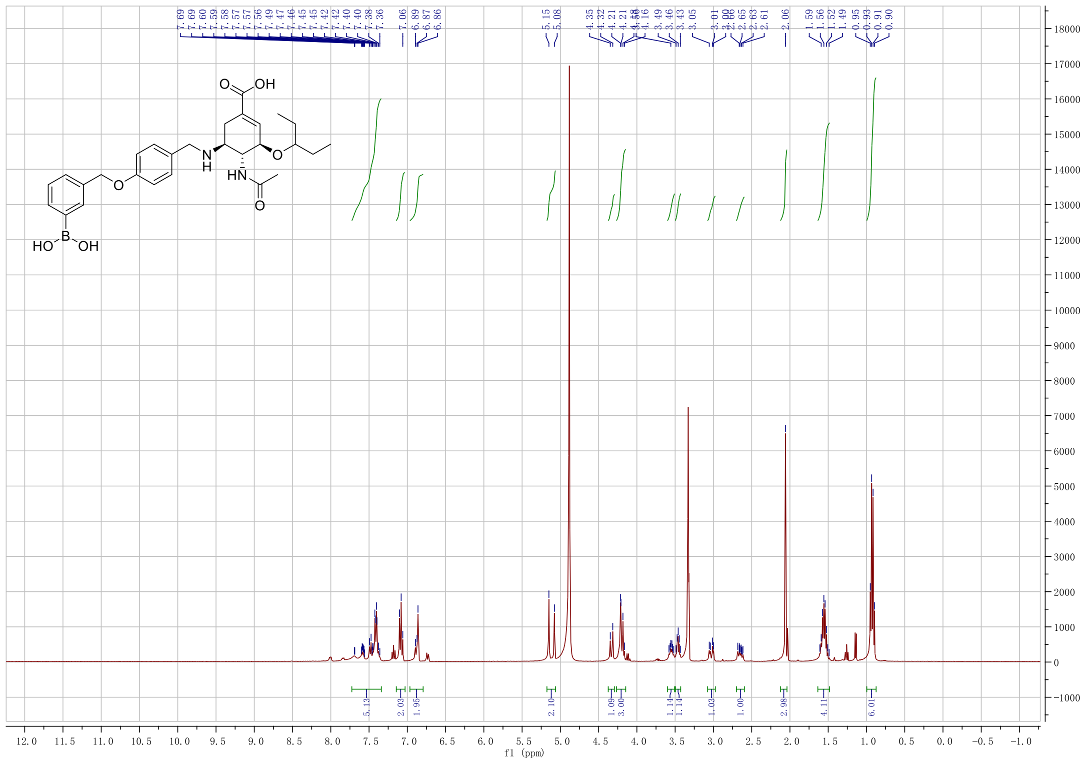

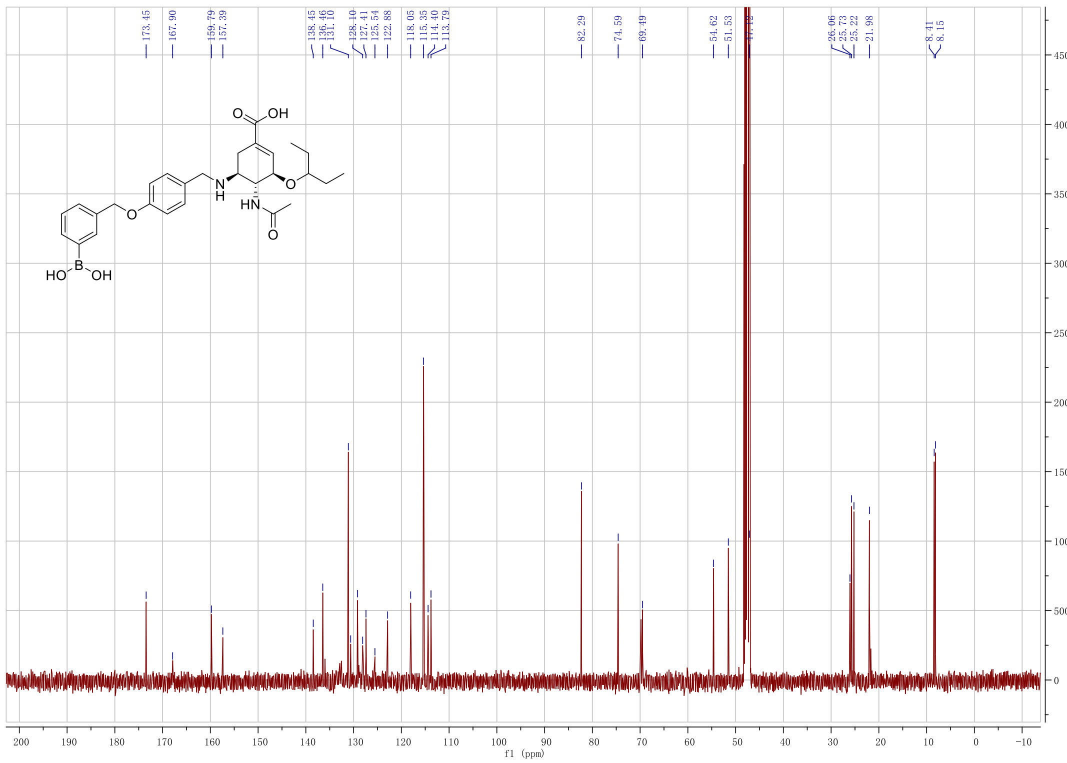

# The $^{1}$H NMR and $^{13}$C NMR spectra of compound 3c

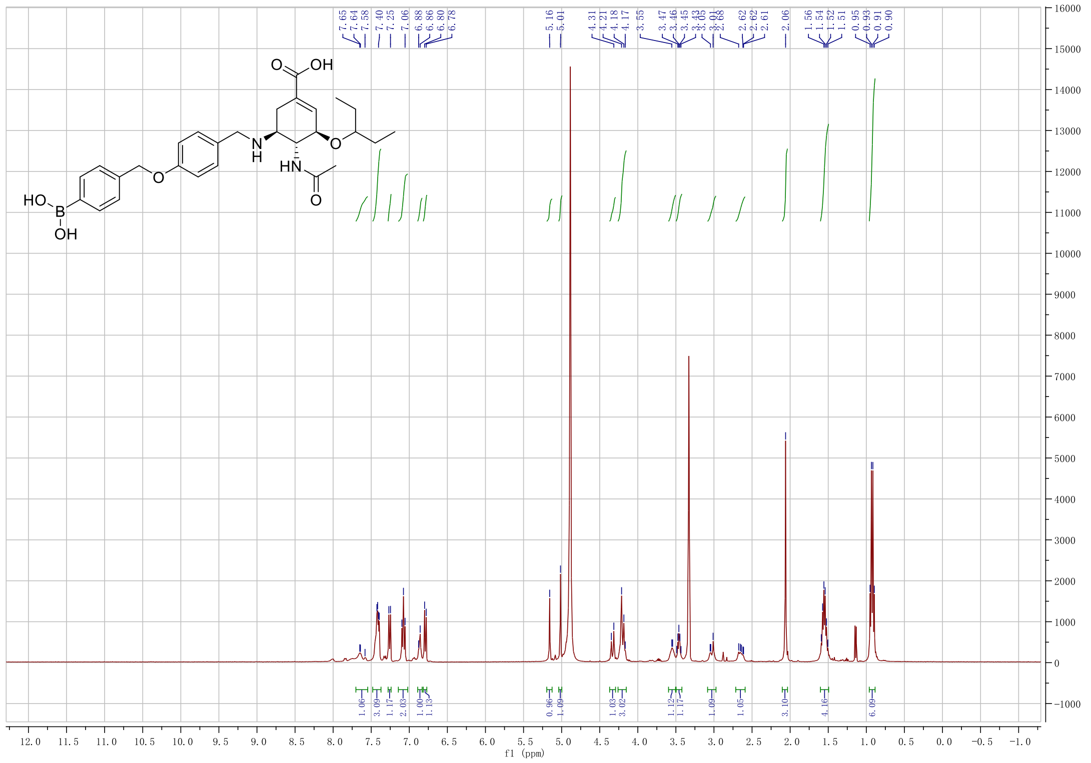


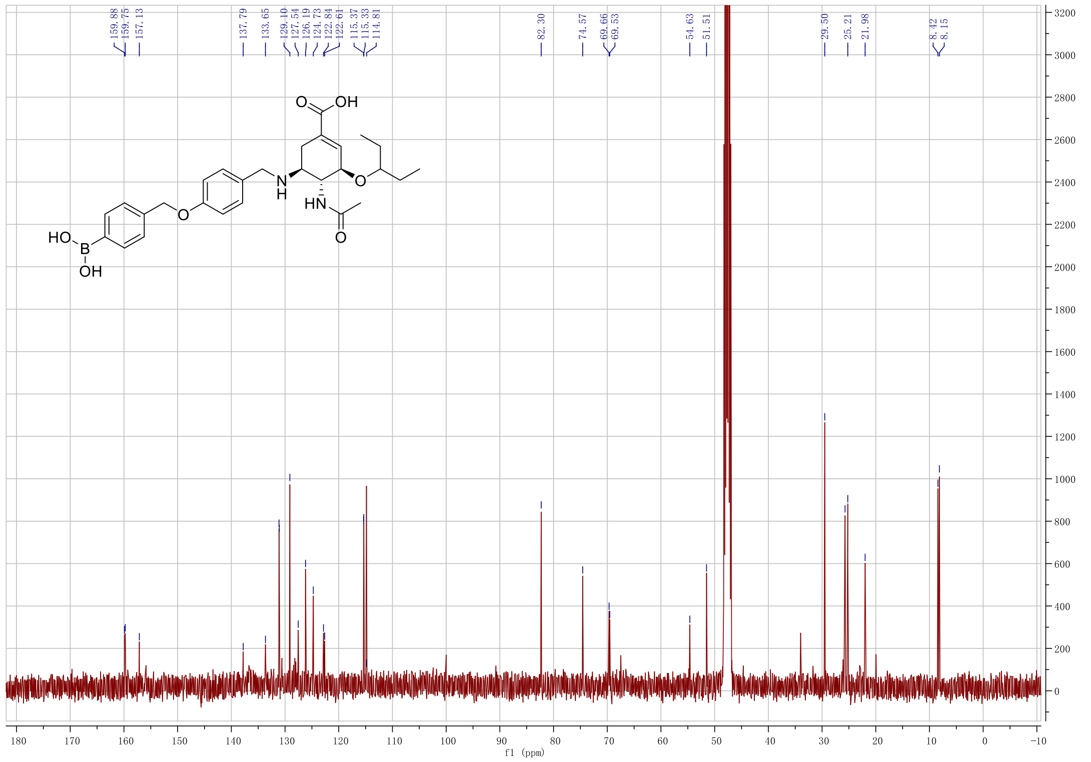

# The $^{1}$H NMR and $^{13}$C NMR spectra of compound 4c

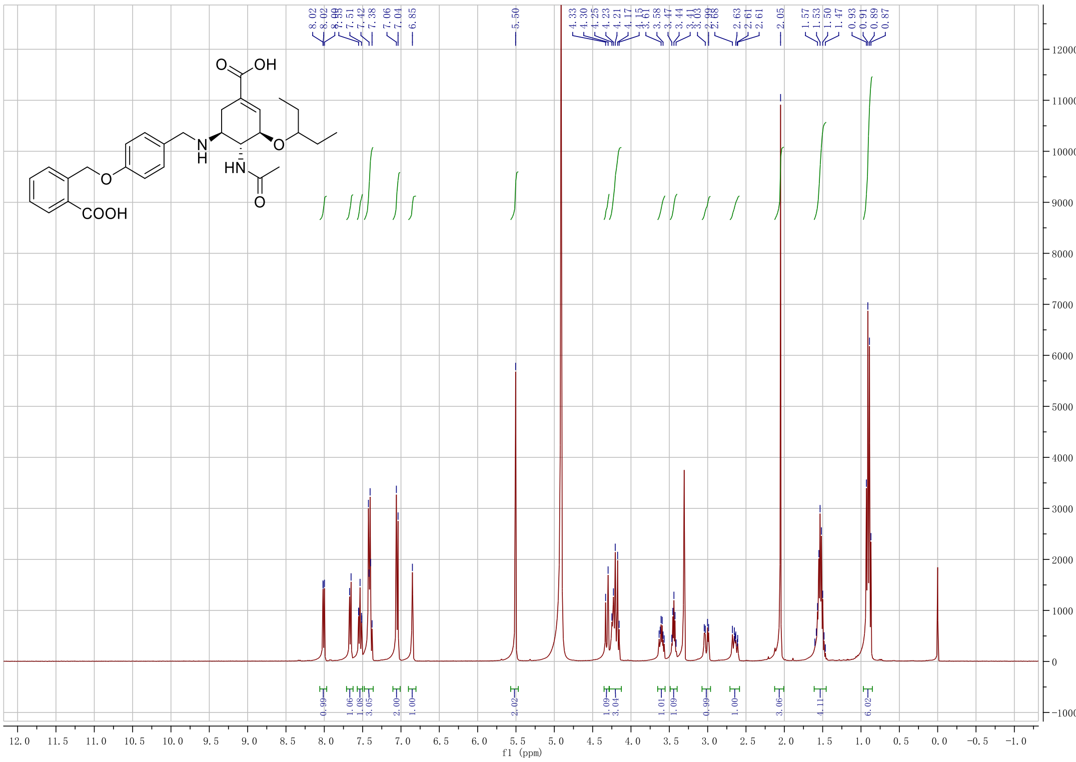


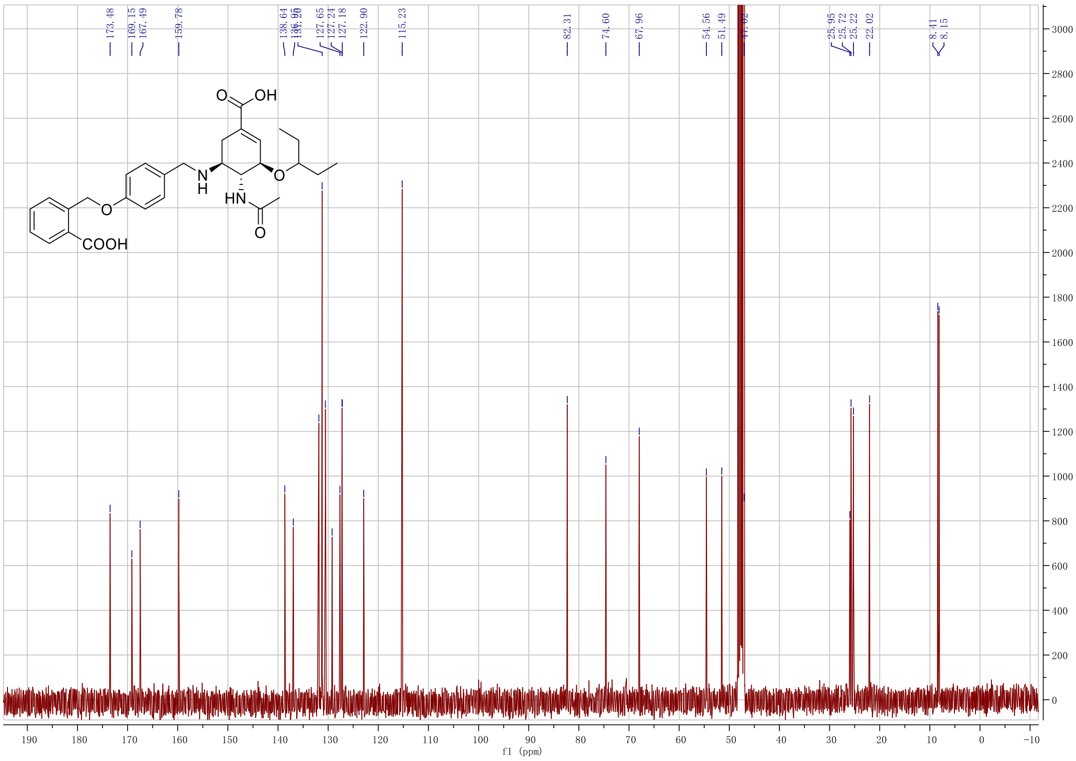

# The $^{1}$H NMR and $^{13}$C NMR spectra of compound 5c

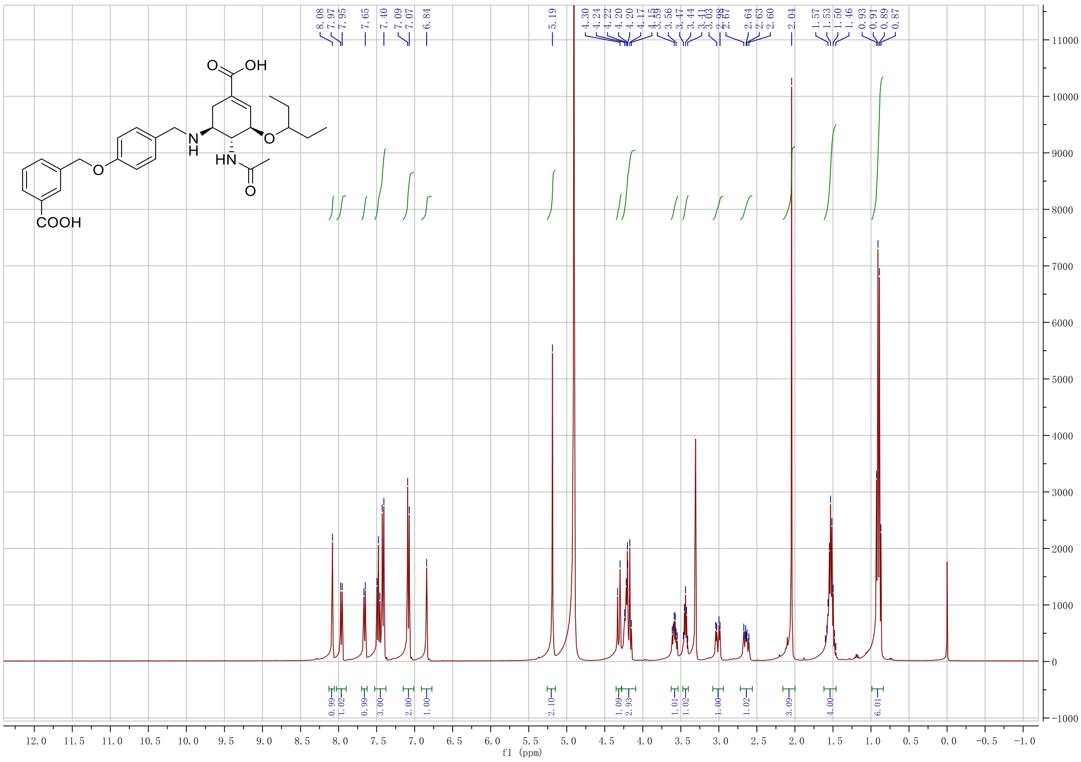


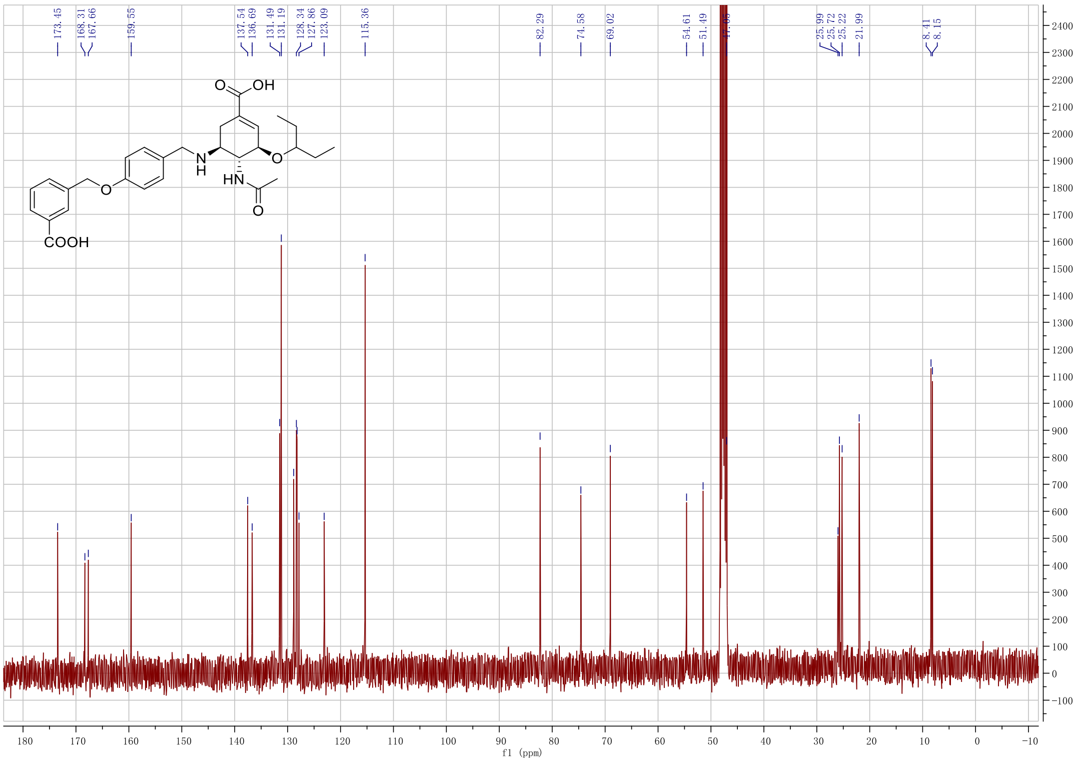

# The $^{1}H$ NMR and $^{13}C$ NMR spectra of compound 6c

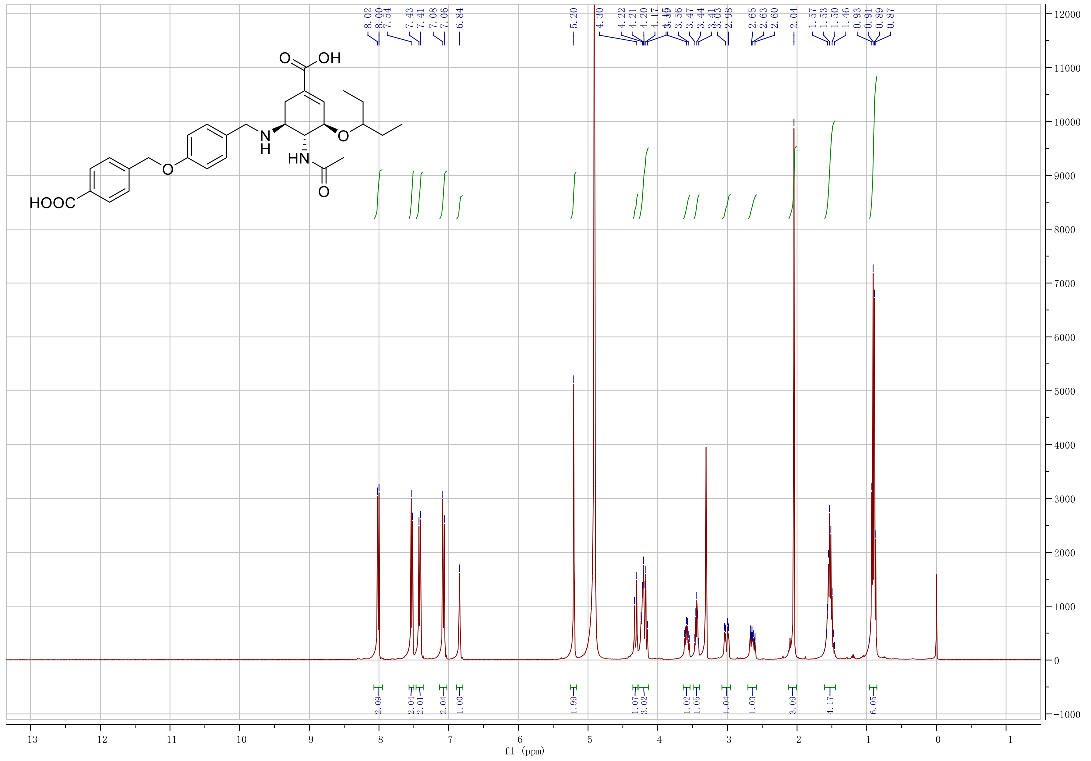

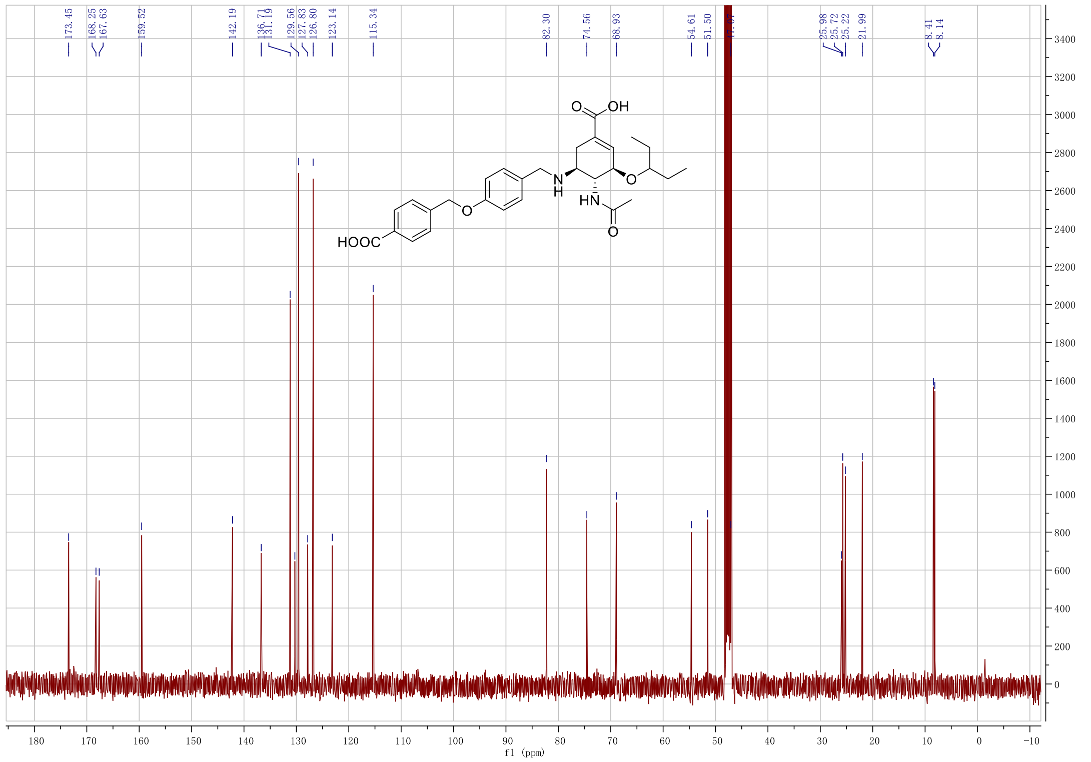

# The $^{1}$H NMR and $^{13}$C NMR spectra of compound 7c

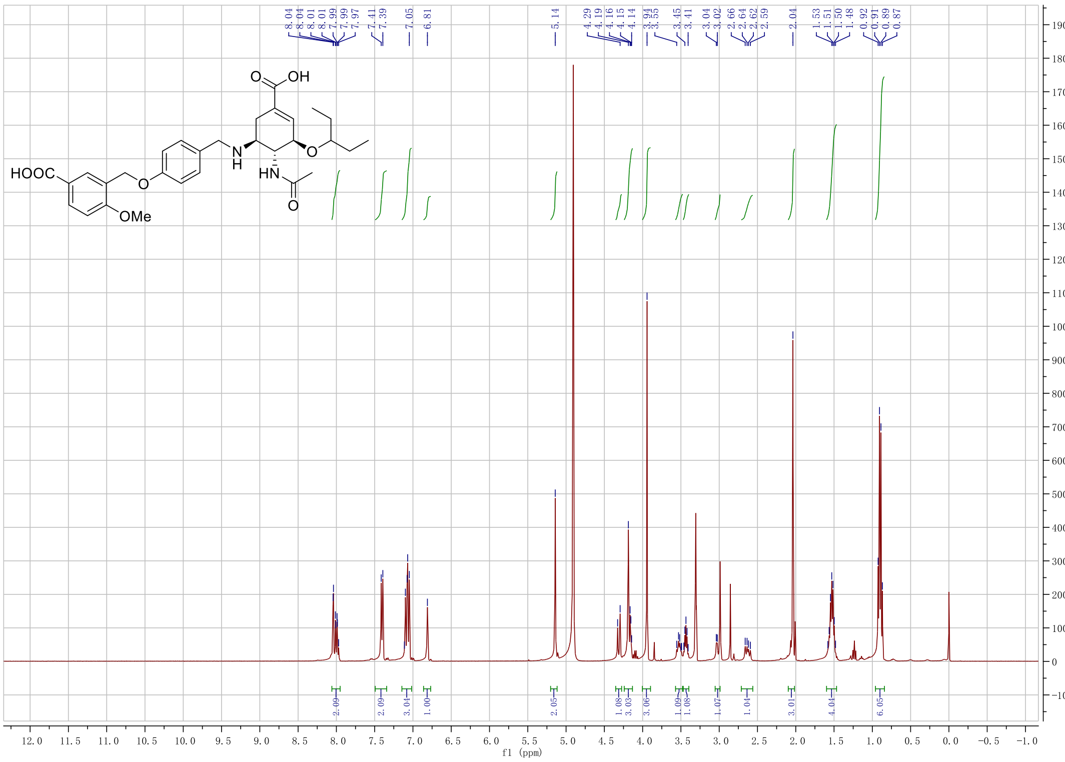


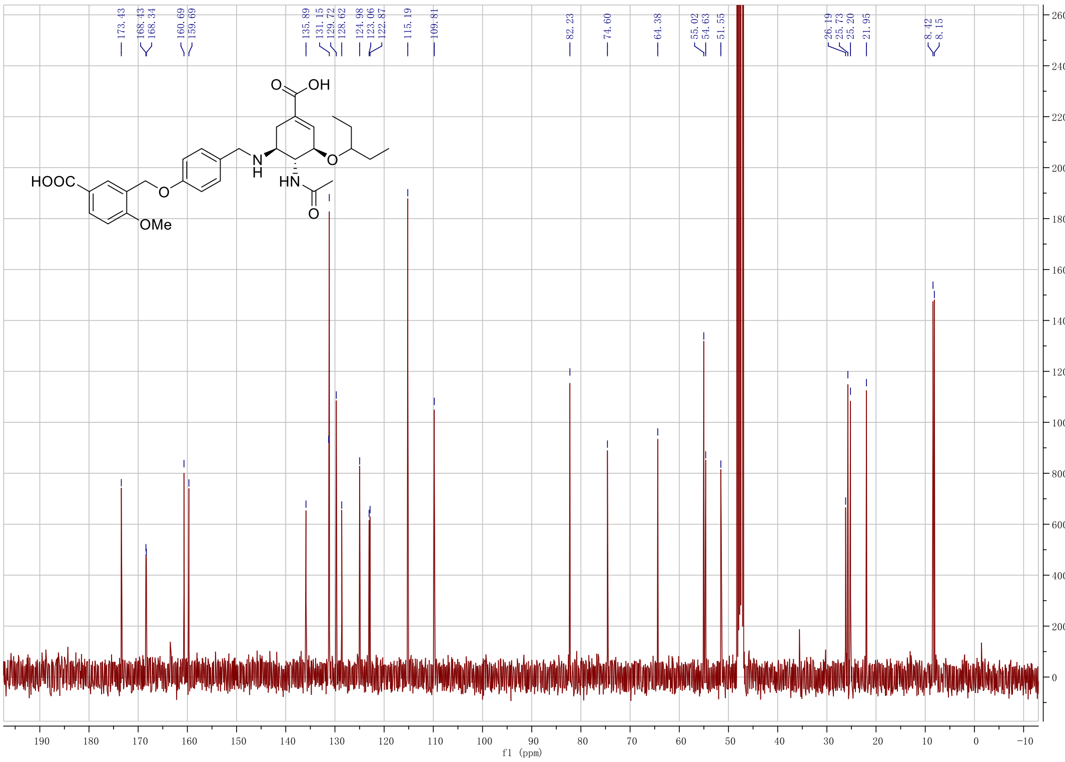

# The $^{1}H$ NMR and $^{13}C$ NMR spectra of compound 8c

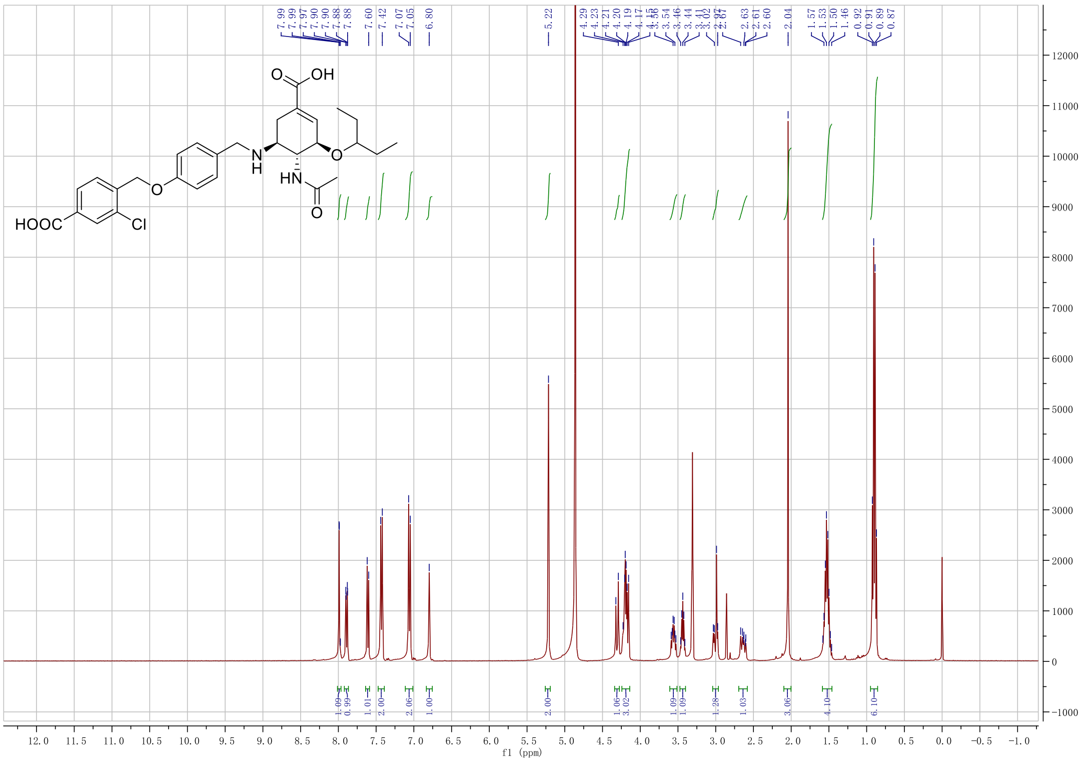

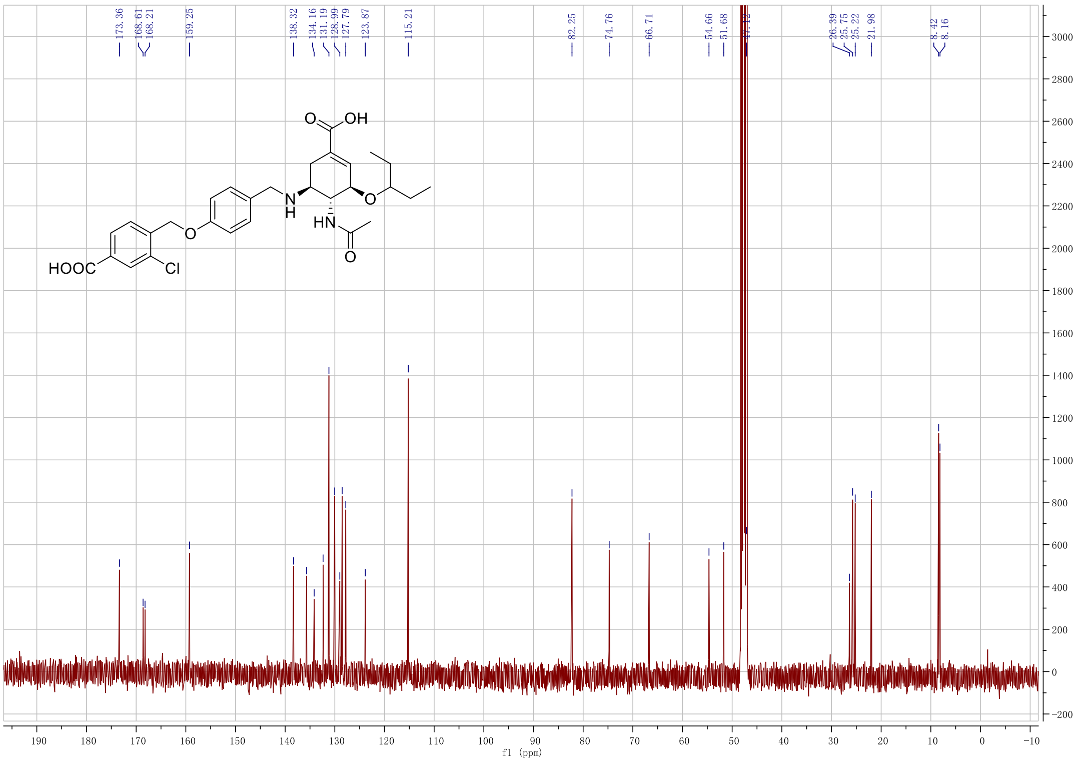

# The $^{1}$H NMR and $^{13}$C NMR spectra of compound 9c

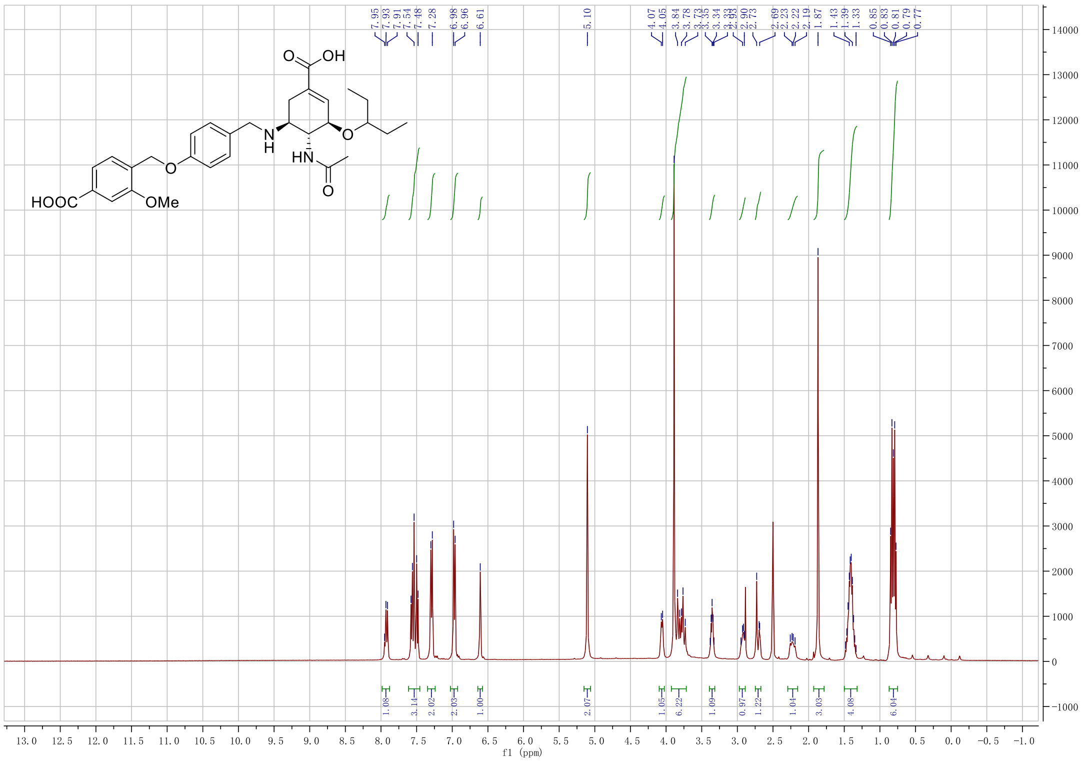


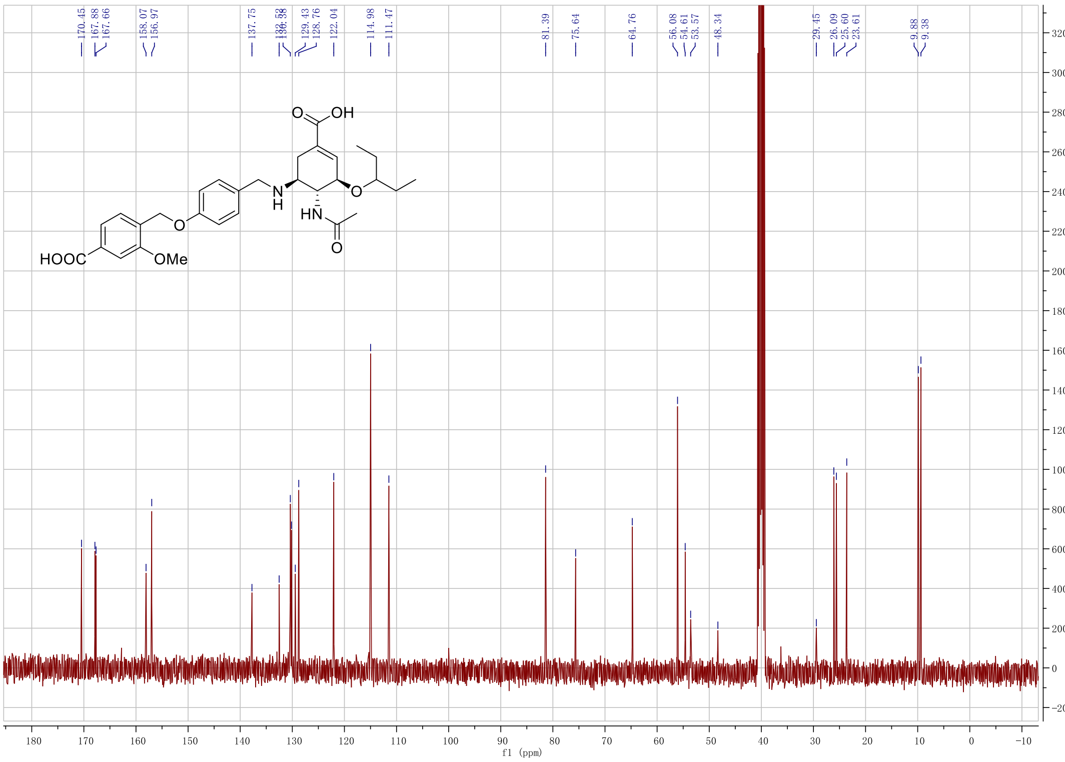

# The $^{1}H$ NMR and $^{13}C$ NMR spectra of compound 10c

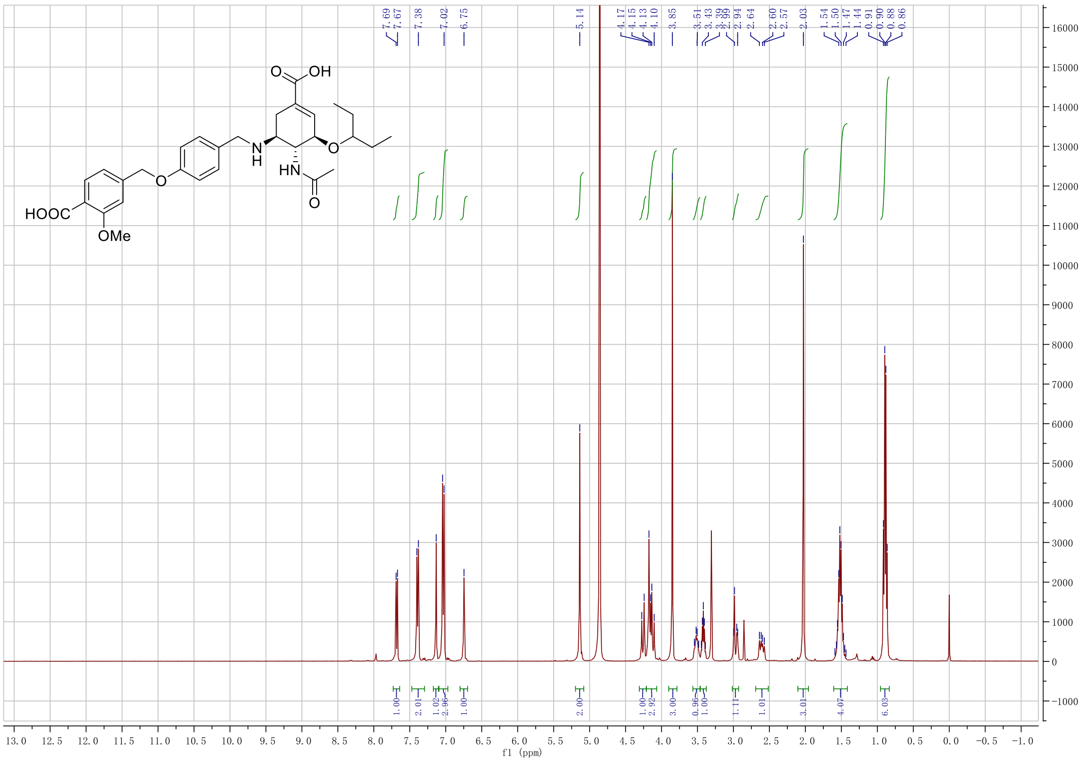

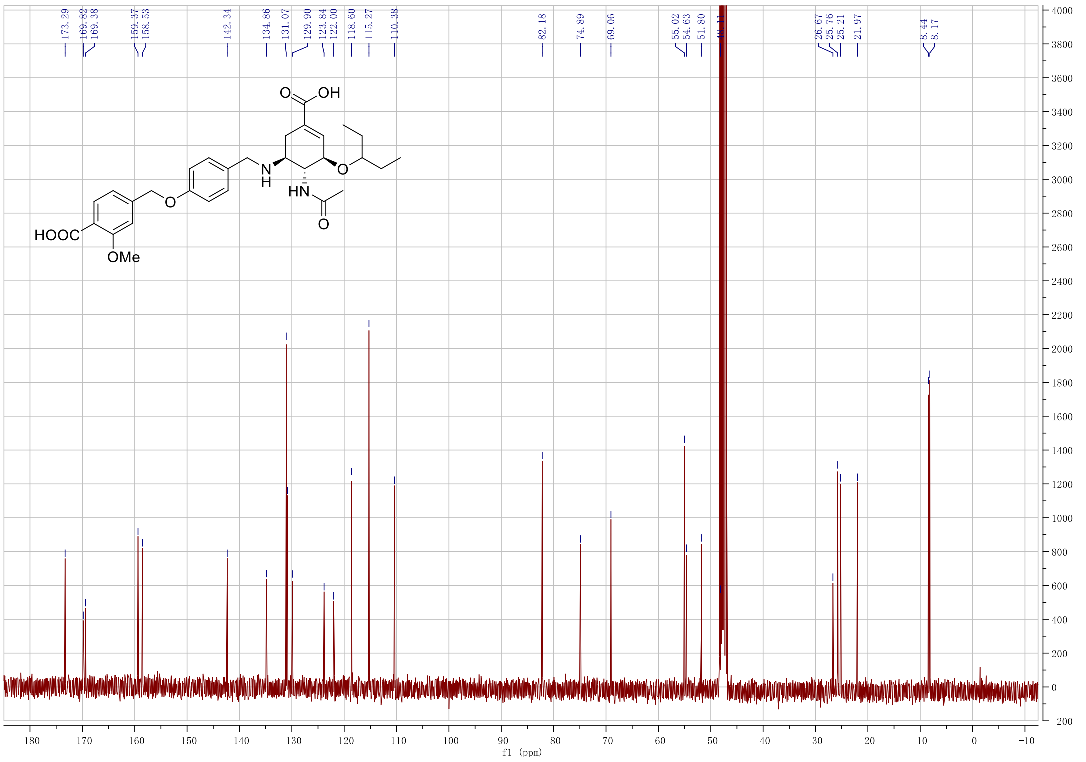

# The $^{1}H$ NMR and $^{13}C$ NMR spectra of compound 11c

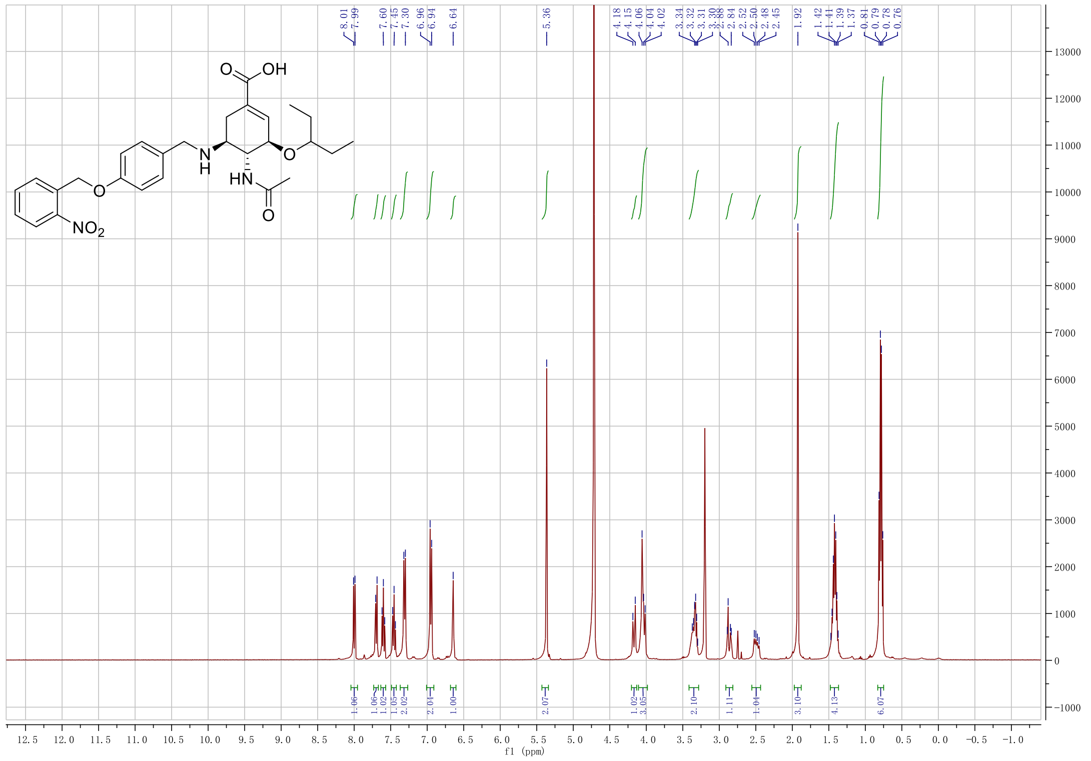

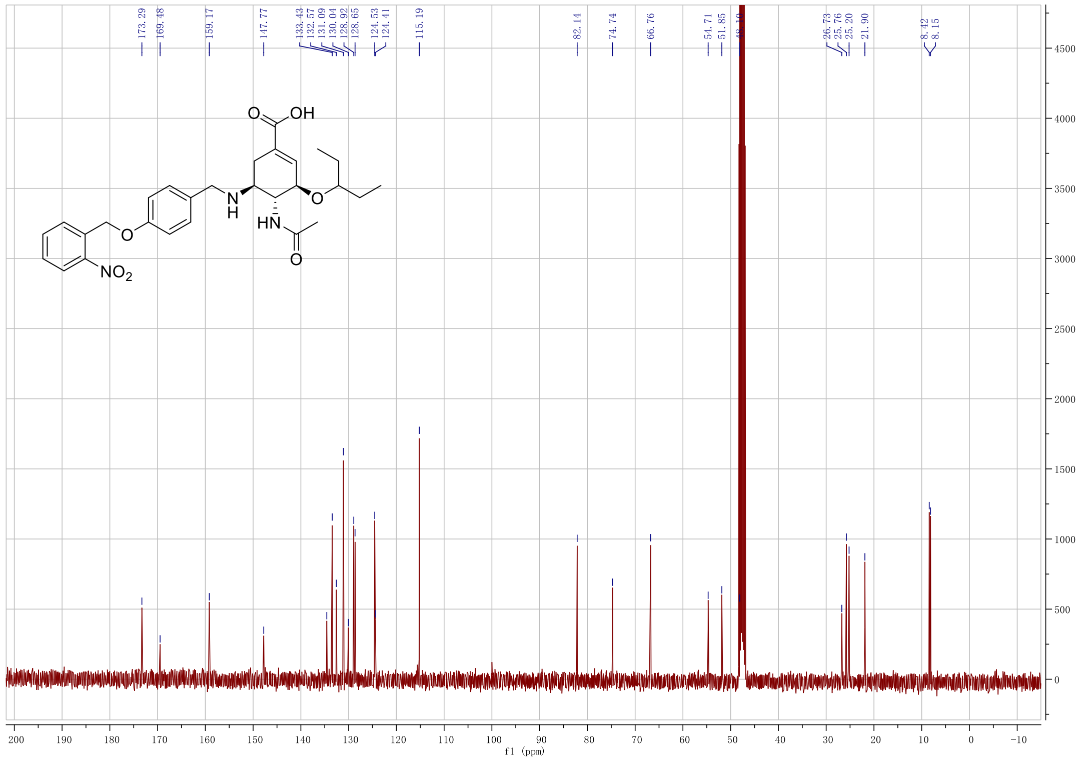

# The $^{1}$H NMR and $^{13}$C NMR spectra of compound 12c

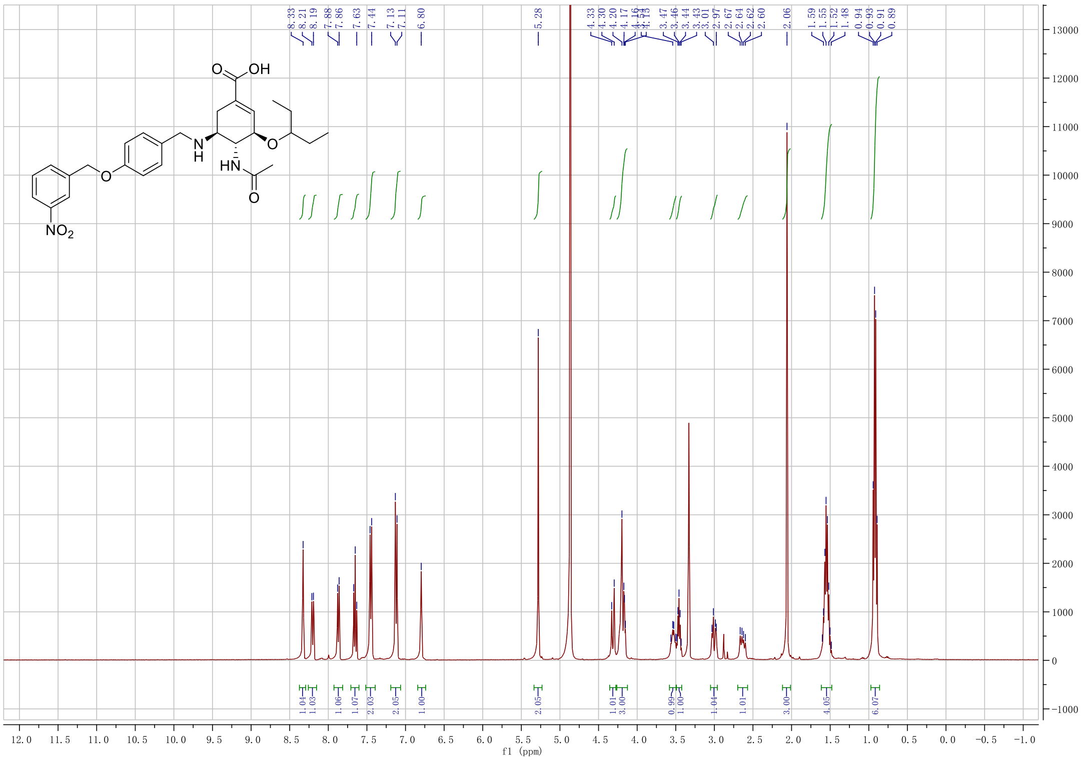

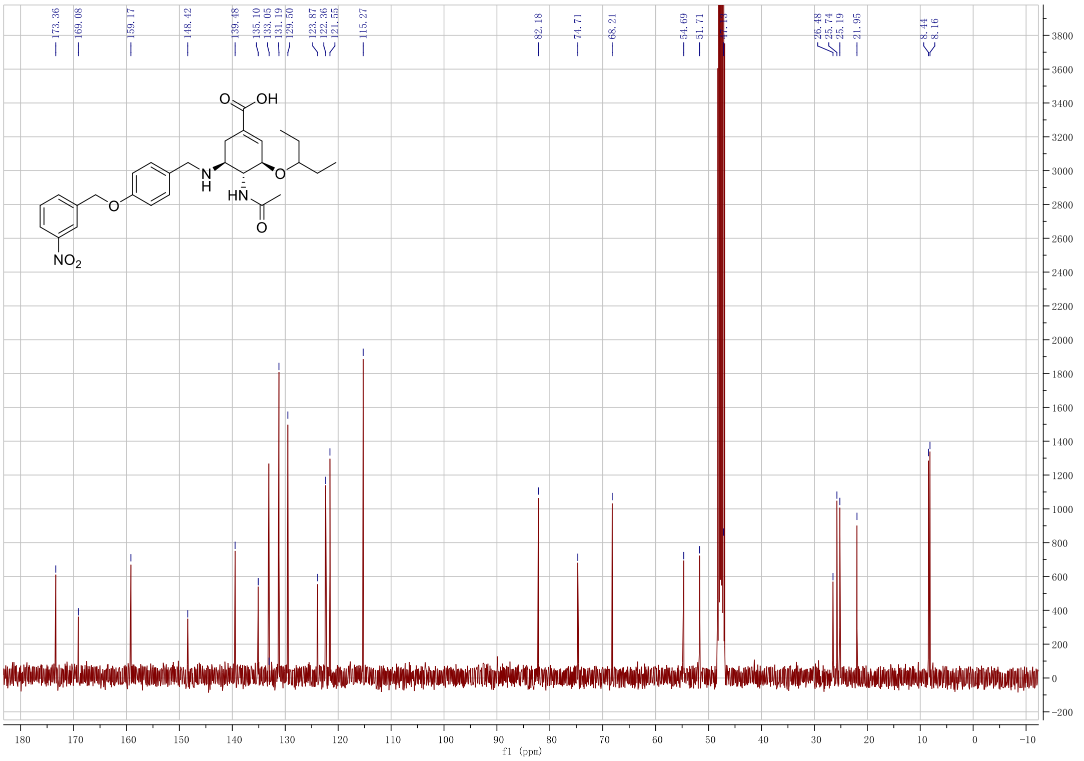

# The $^{1}$H NMR and $^{13}$C NMR spectra of compound 13c

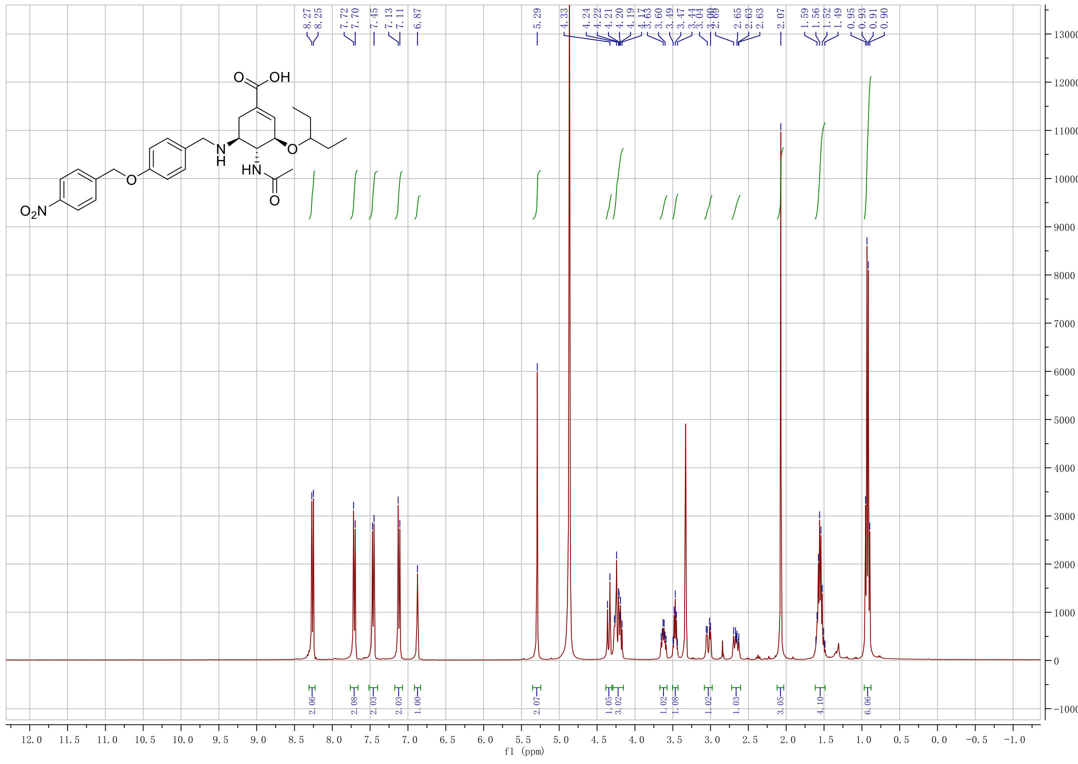

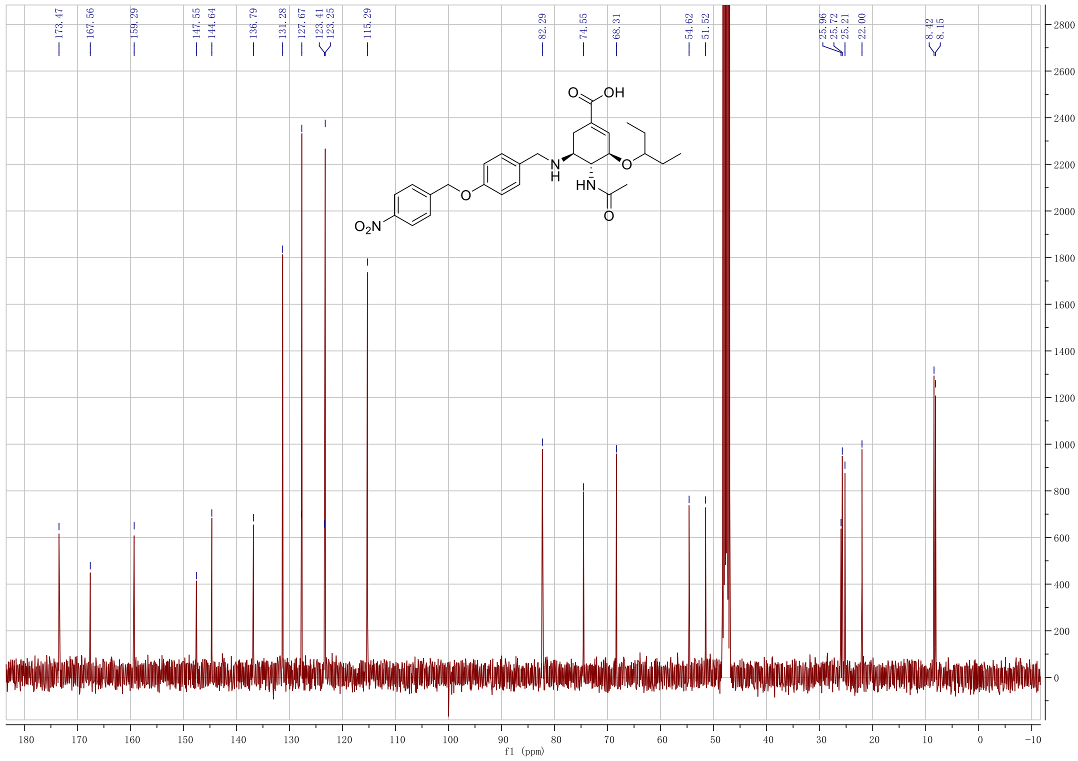

**The $^{1}$H NMR and $^{13}$C NMR spectra of compound 14c**

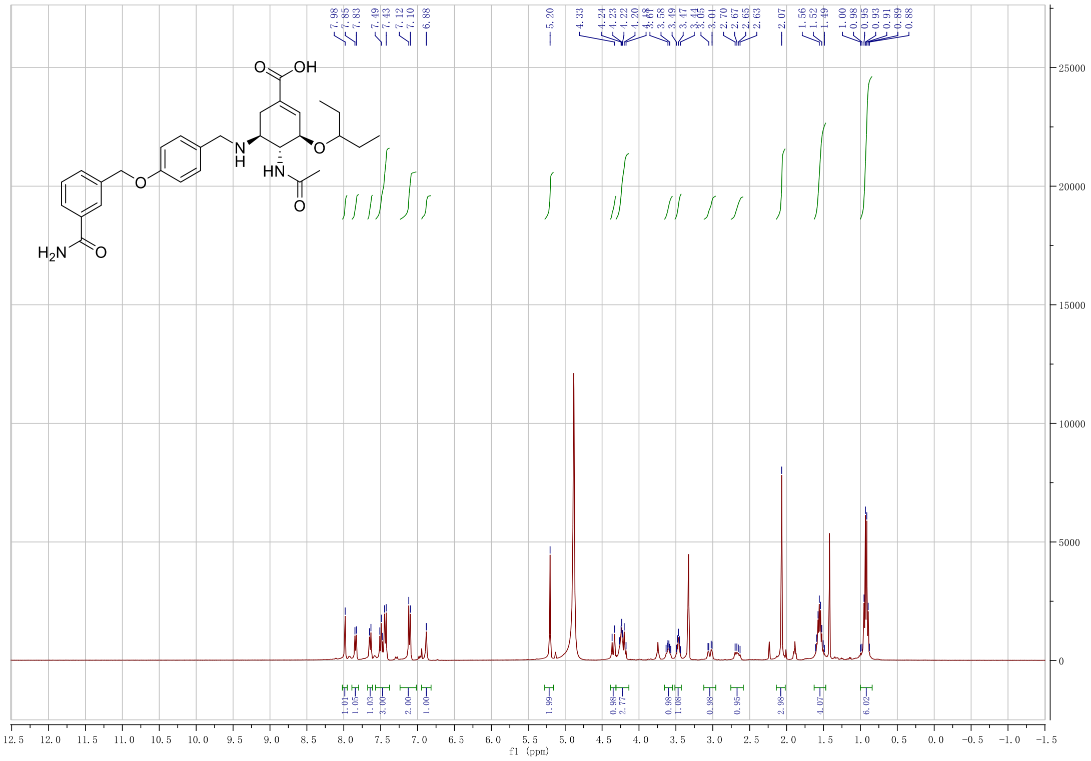

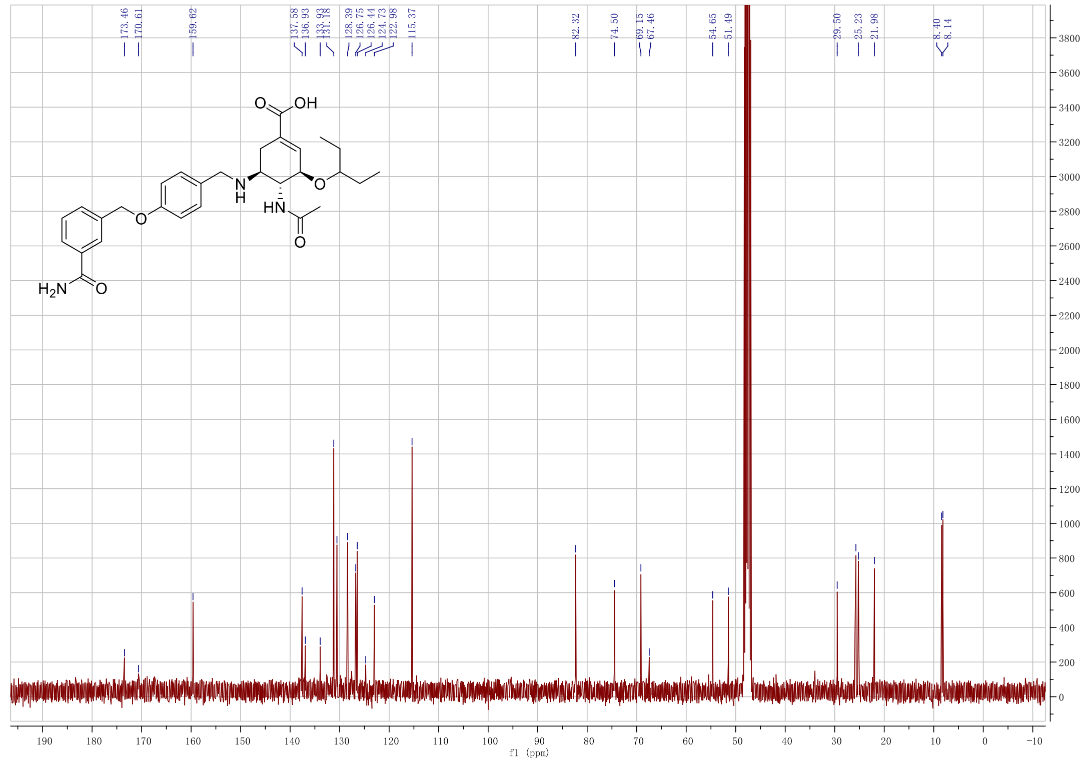

# The $^{1}$H NMR and $^{13}$C NMR spectra of compound 15c

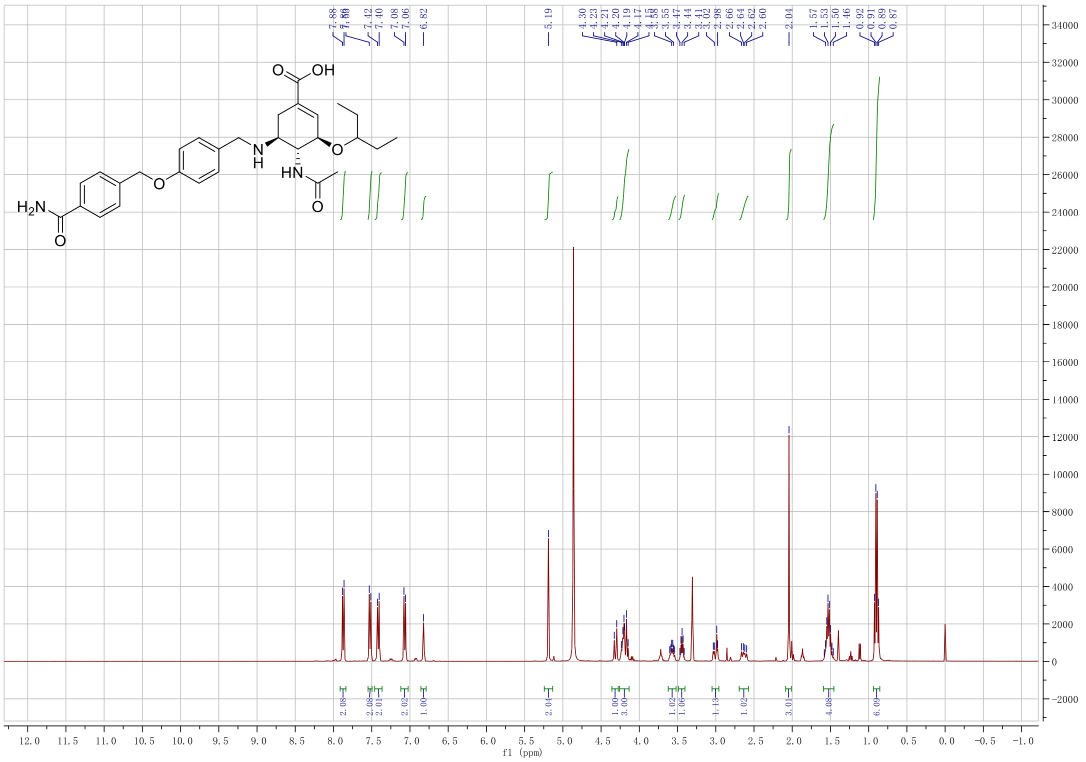

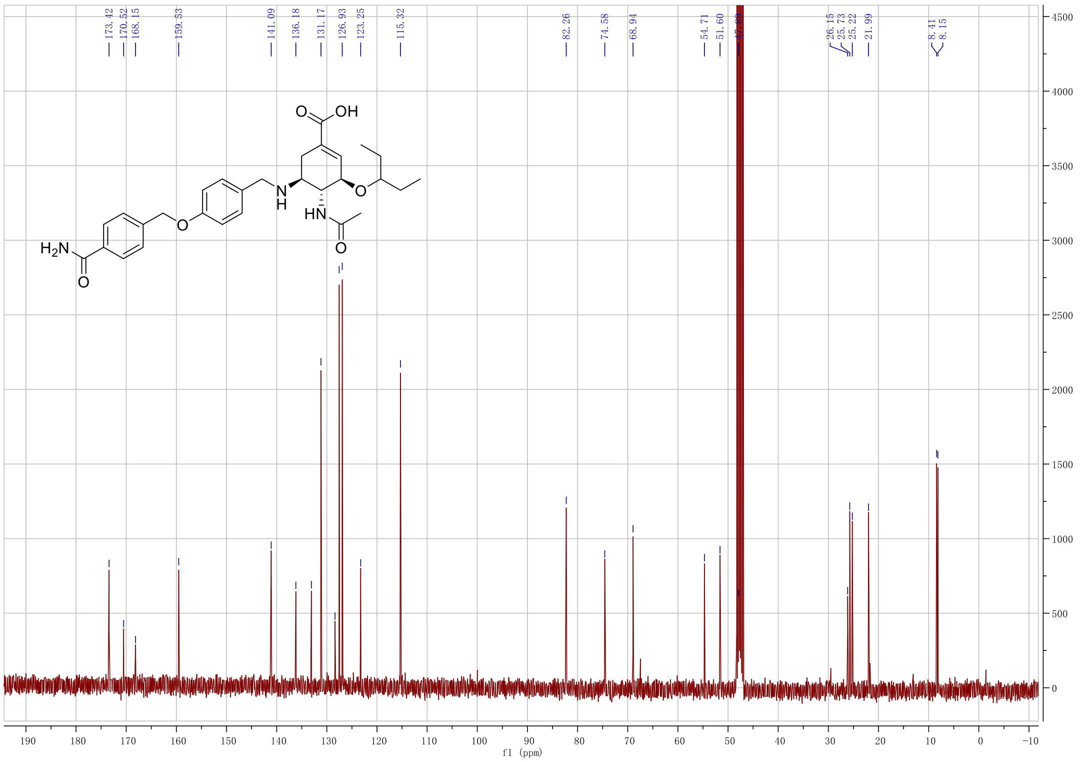

# The $^{1}H$ NMR and $^{13}C$ NMR spectra of compound 16c

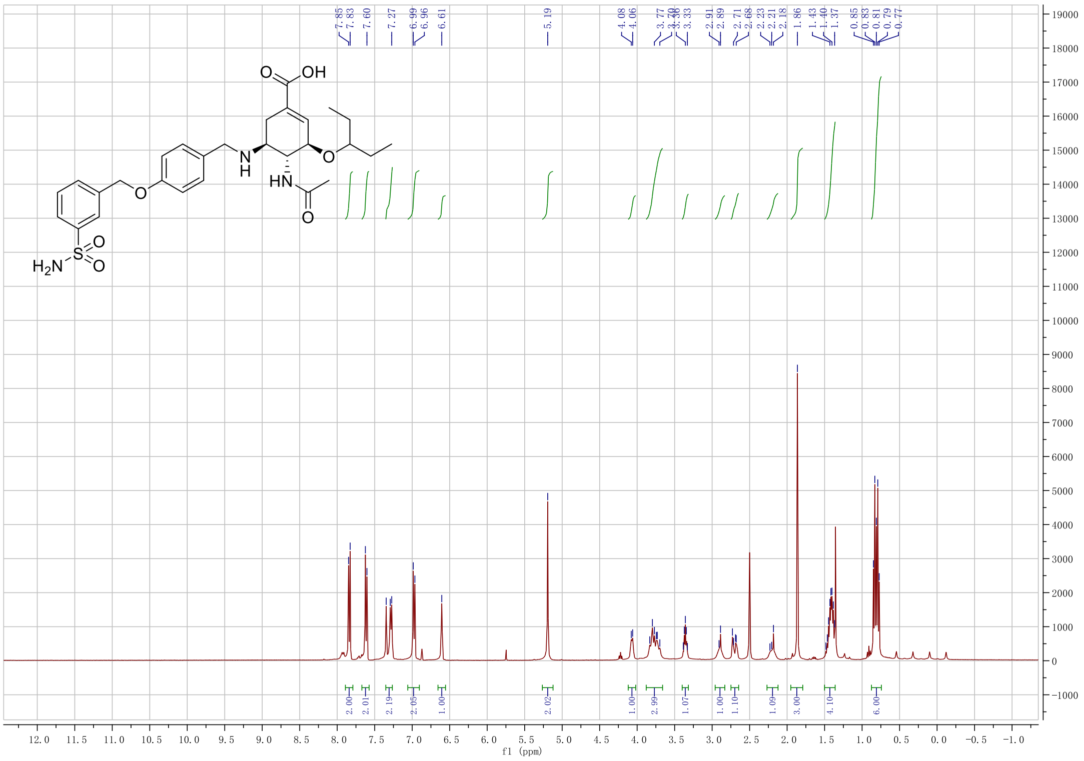


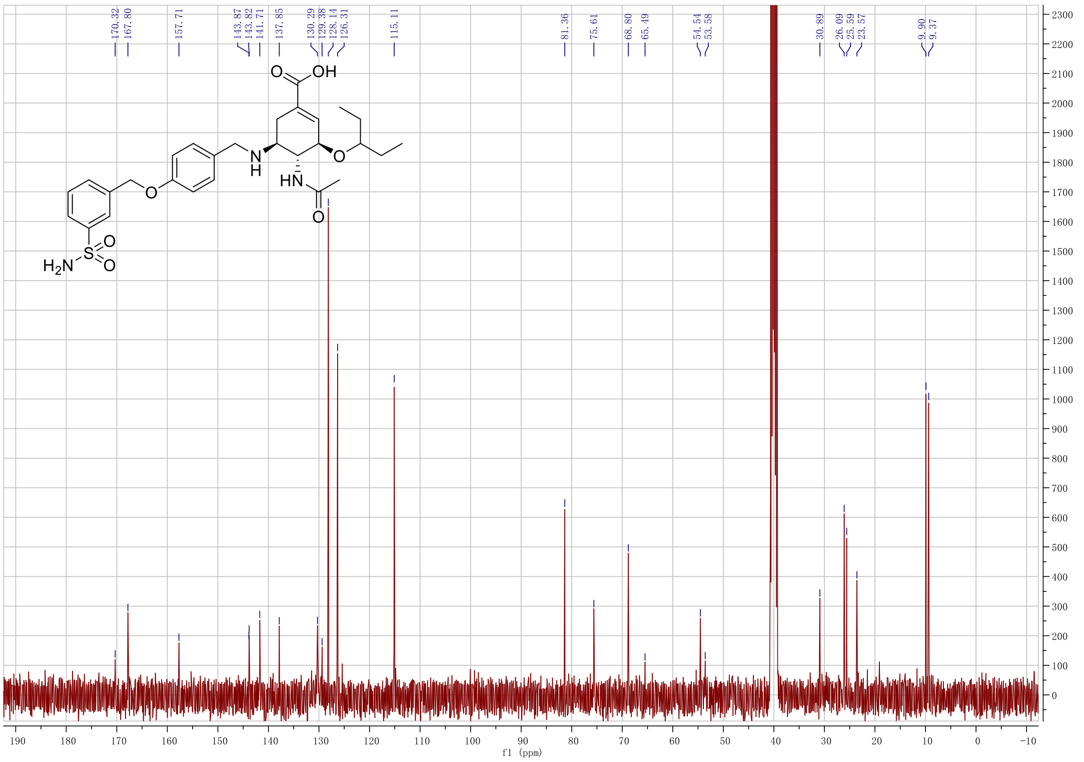

# The $^{1}$H NMR and $^{13}$C NMR spectra of compound 17c

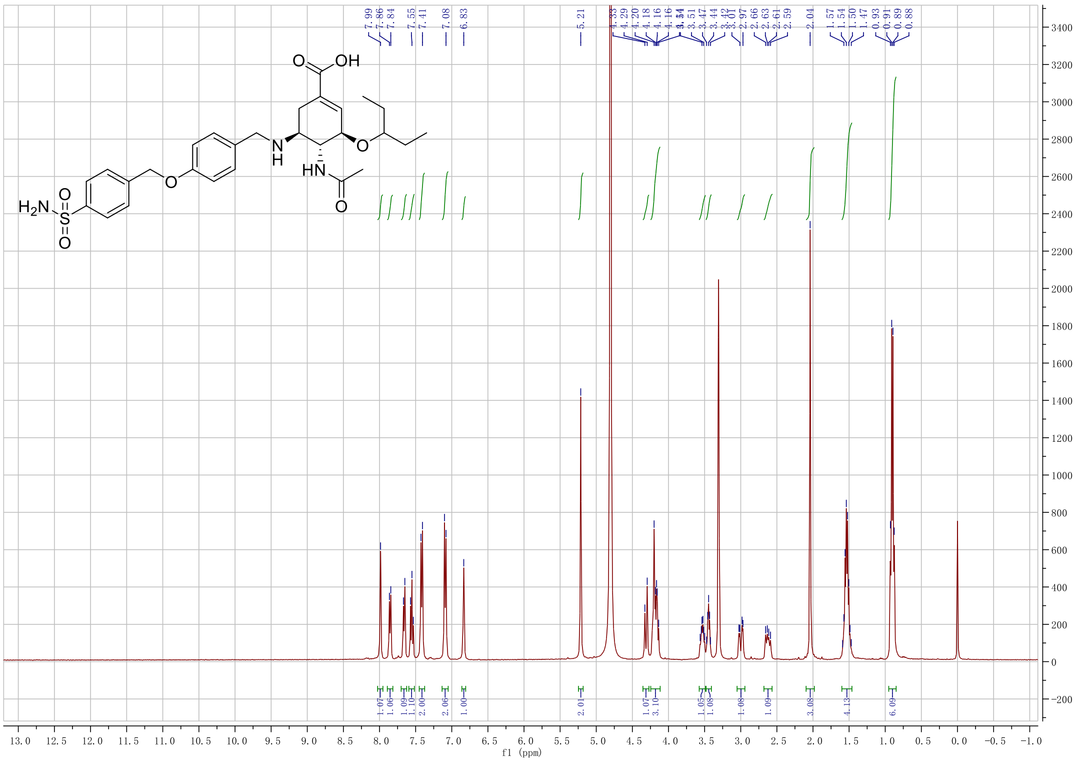

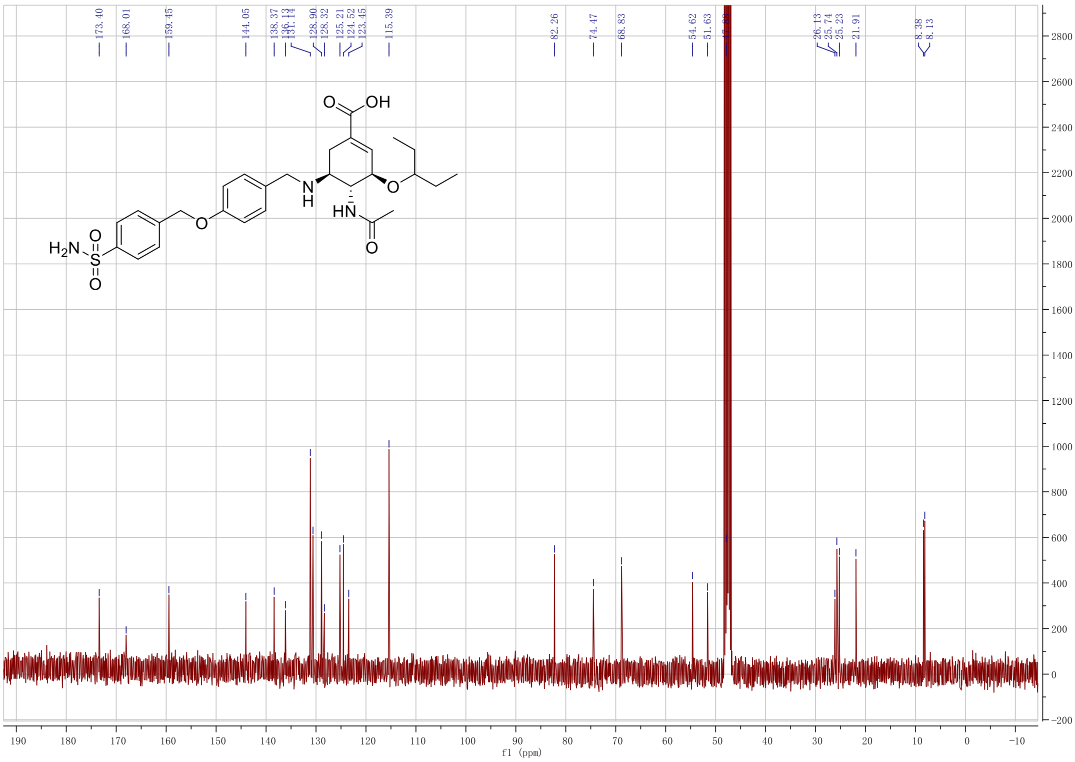

# The $^{1}$H NMR and $^{13}$C NMR spectra of compound 18c

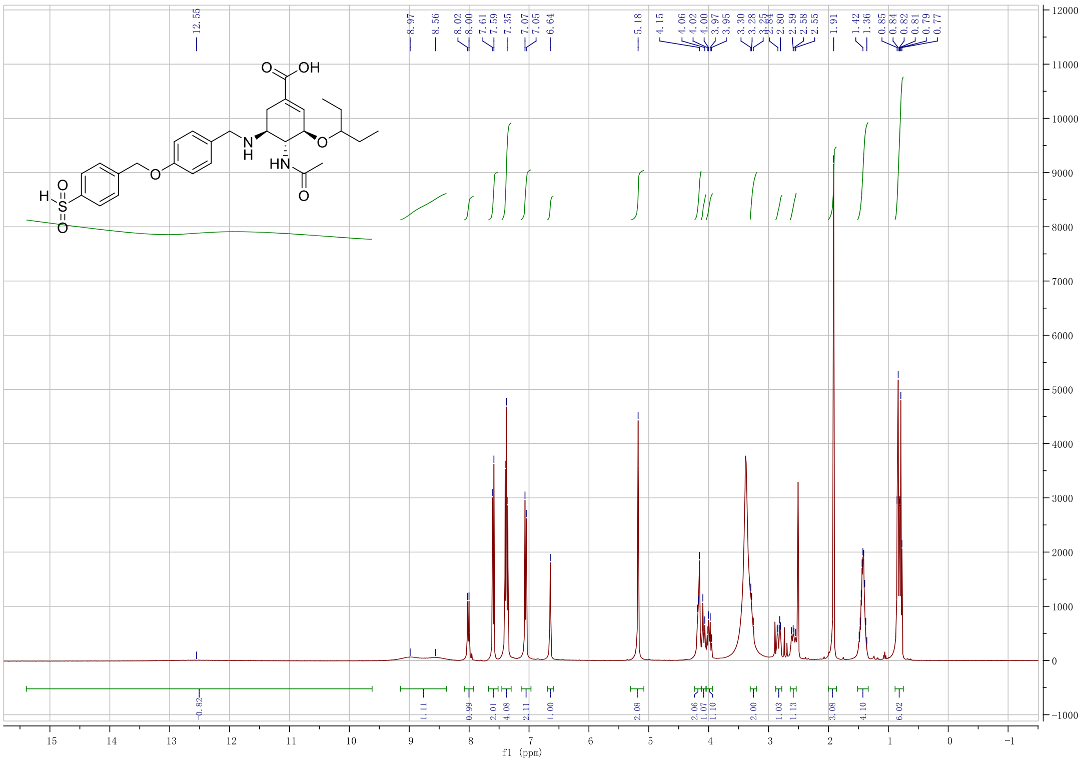

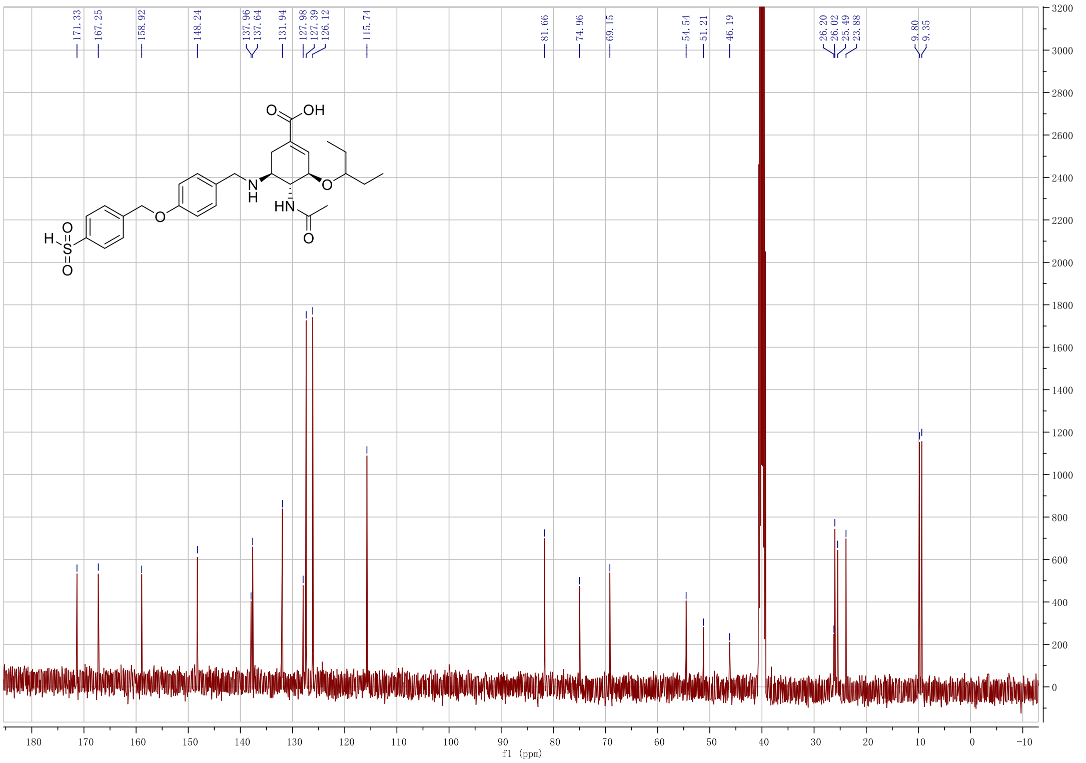

Supplement: Supplementary file 1 [file molecules-27-06426-s001.zip › Supplementary Material.pdf]
